# CAR-macrophages: tailoring cancer immunotherapy

**DOI:** 10.3389/fimmu.2024.1532833

**Published:** 2025-01-14

**Authors:** Dewan Chettri, Bibhu Prasad Satapathy, Rohit Yadav, Vivek Uttam, Aklank Jain, Hridayesh Prakash

**Affiliations:** ^1^ Non-Coding RNA and Cancer Biology Laboratory, Department of Zoology, Central University of Punjab, Bathinda, India; ^2^ Amity Centre for Translational Research, Amity University, Noida, India

**Keywords:** CAR macrophage, immune mediated rejection, immune homeostasis, tumor killing, Th1 response

## Background

The ability of effector immune cells to target and eliminate tumor cells by focusing on tumor-associated antigens is crucial for the success of immunotherapy. Chimeric Antigen Receptor (CAR)-modified immune cells have revolutionized cancer immunotherapy, primarily with CAR-T cells showing remarkable success in hematological cancers. Numerous cell-based therapies, such as CAR-T cell, TIL, CAR-NK cell, and T-cell receptor (TCR)-based therapies, are currently undergoing clinical and pre-clinical evaluation across various cancer types. However, these cell-based therapies possess limitations, including their inability to penetrate tumor stoma, change TME, and exaggerated inflammatory responses. To overcome this, developing advanced and flexible strategies to target tumor cells precisely while preserving immune homeostasis in cancer patients is imperative. One promising approach involves using engineered tumorassociated macrophages (TAMs), which are plastic in nature and constitute approximately 50% of the tumor microenvironment (TME) and are indispensable for both tumor progression and regression. The engineered CAR-macrophages aim to reprogram macrophages toward the M1 TAM phenotype and enable them to overcome the immune-suppressive TME and facilitate immune-mediated destruction of tumors. The tumor microenvironment (TME) typically comprises various immune cells, including tumor-associated macrophages (TAMs), which is one of the major components of TME and play crucial role in cancer progression or regression (1). Tumor-associated macrophages are extremely plastic in nature and due to this unique property these cells both promote as well as control the tumor development which depend on the predominating phenotype established. TAMs within the TME can be identified as either M1 TAMs (regulatory) and / or M2 TAMs (trophic). Interestingly in variety of tumor patients, these phenotypes remain in dynamic equilibrium (2). During neoplasia, tumor infiltrating TAM acquire M1 phenotype where these cells, by virtue of their Th1 effector responses, are able to control the tumor development. However, during later phase of tumor development, tumor infiltrating M1 TAM, under influence of refractory tumor microenvironment, get polarized toward M2 TAM (3–5). These are potentially trophic in nature and secrete various pro-tumoral and angiogenic factors, such as IL-10, TGF-β, and VEGF, contributing to an immunosuppressive environment that favors tumor growth (6). M2 TAM confer poor prognosis in large variety of tumor patients (7) therefore retuning of M2 TAM toward M1 TAW is pre-requisite of effective immune therapy of variety of tumors. Various efforts are under progress for engineering macrophages to express receptors that recognize vide range of tumor-associated antigens (TAA) are in the pipeline, allowing them to selectively target and destroy cancer cells. These engineered macrophages, known as Chimeric Antigen Receptor Macrophages (CAR-Macrophages) are capable enough of priming of tumor reactive T-cells while keeping their natural tumor-targeting ability (8). Thus, it is clear that CAR-Macrophage (CAR-M) therapy would be potential asset of immunotherapy for many solid tumors.

## Underlying mechanism

**Figure 1:**
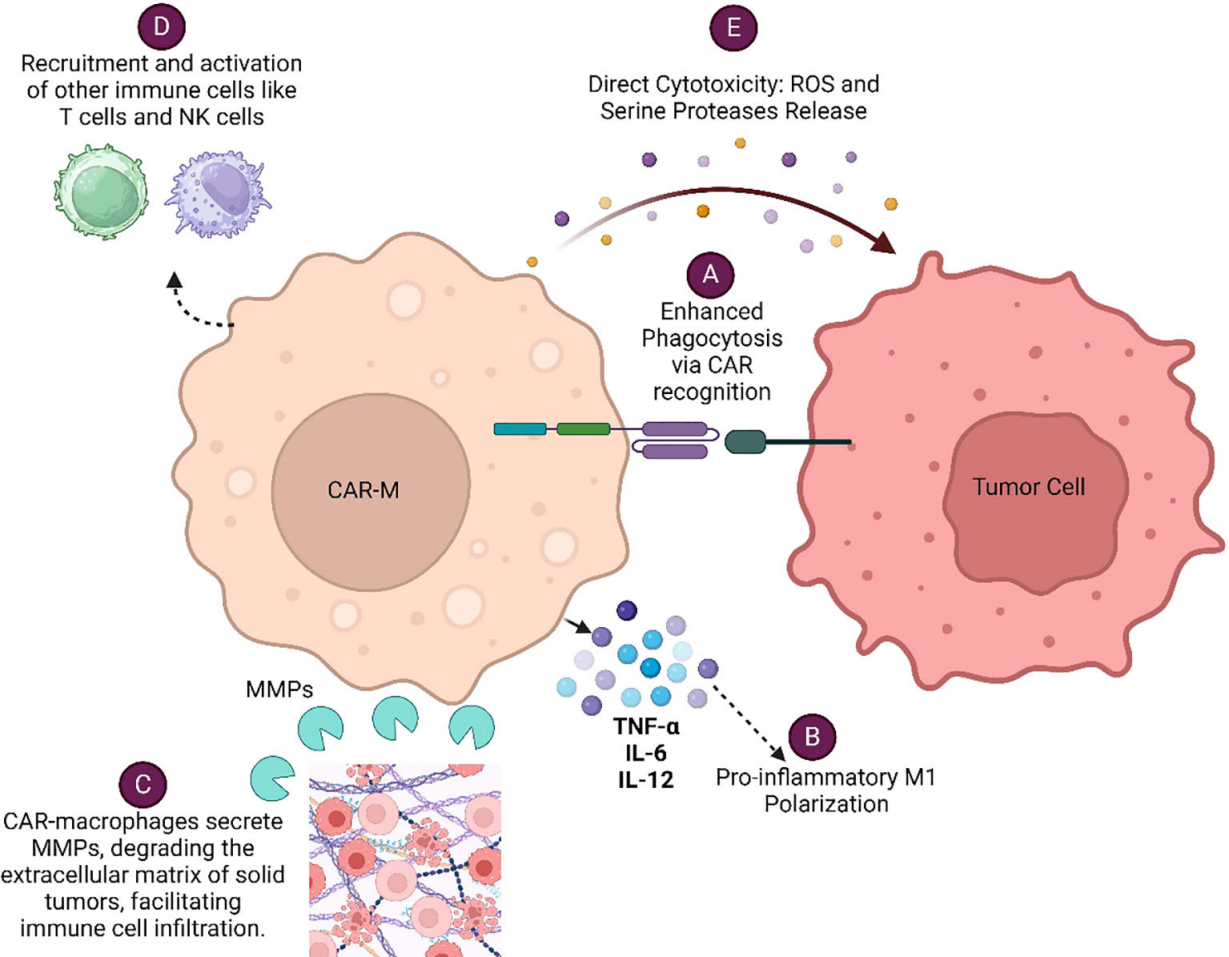
Various mechanisms by which CAR-Macrophages (CAR-Ms) eliminate cancer cells: **(A)** Enhanced Phagocytosis via CAR Recognition **(B)** Pro-inflammatory M1 Polarization **(C)** Modulation of Tumor Microenvironment **(D)** Recruitment and Activation of Other Immune Cells **(E)** Direct Cytotoxicity.

CAR-Macrophage (CAR-M) recognizes specific tumor antigens through its chimeric antigen receptor (CAR), leading to enhanced phagocytosis of the tumor cells and concomitant presentation of tumor antigen to T cells for their subsequent activation. CAR-Ms modulate tumor microenvironment by secreting various cytokines, metalloproteinases (MMPs), Reactive oxygen species (ROS) and serine proteases which altogether facilitate immune cell infiltration and enhanced anti-tumor responses of immune cells. Each of these mechanisms (**Figure 1**) contributes to the overall effectiveness of CAR-Macrophages in targeting and eliminating cancer cells, making them a promising therapeutic approach in cancer immunotherapy. CAR-engineered macrophages were shown to actively migrate to tumor sites, could locally deliver cytokines and/or cytotoxic substances to antigen-specific environments when used as a drug delivery system, significantly alter the immune-suppressive TME and eliminate tumor cells through phagocytosis (9). These strategies leverage the tumor-homing tendencies of macrophages to locally deliver therapeutic cargo and induce cytotoxic activity within the tumor niche.

With the help of regenerative technologies, induced pluripotent stem cells (iPSCs) can be engineered to make CD19/mesothelin+ CAR-Ms which are M1^programmed^ and bear strong anti-tumoral potential (10). Engineered macrophages can be derived from diverse sources, including primary human monocytes/macrophages, induced pluripotent stem cells (iPSCs), and hematopoietic stem and progenitor cells (HSPCs). Abdin et al. (2023) used iPSCs, HSPCs to generate anti-CD19 CAR-Ms. They generated αCD19 CAR constructs using lentiviral vectors verified via sequencing. CD34+ cells were isolated from cord blood, transduced, and differentiated into macrophages. iPSCs were cultured, mesoderm-primed, and differentiated into macrophages using defined cytokines. Cancer cell lines and patient-derived samples were transduced and co-cultured with CAR macrophages to assess phagocytosis. Then they used flow cytometry, confocal microscopy, and western blotting to verify the action of CAR-Ms. CAR-Ms displayed antigen-specific phagocytosis of CD19+ cancer cells, enhanced pro-inflammatory responses, and adaptive immune cell recruitment, with scRNA sequencing revealed distinct activation of pro-inflammatory pathways and cytokine upregulation (11). In another study by Zhang et al. (2023), they utilized CRISPR-Cas9 gene editing to integrate an anti-GD2 CAR into the AAVS1 locus of human pluripotent stem cells (hPSCs). Then they developed a serum- and feeder-free differentiation protocol to generate CAR macrophages (CAR-Ms) through arterial endothelial-to-hematopoietic transition (EHT). The CAR-Ms generated this way demonstrated potent cytotoxic activity against GD2-expressing neuroblastoma and melanoma *in vitro* and neuroblastoma *in vivo*. It can be a useful protocol for generating off-the-shelf CAR-Ms, advancing antitumor immunotherapy applications (12). To improve the efficiency and functionality of hPSC derived CAR-Ms shen et al. (2024) developed an optimized monolayer-based system for achieving stable CAR expression and strong tumoricidal activity *in vitro*. To address diminished *in vivo* activity, they employed interferon-γ and monophosphoryl lipid-A to induce innate immune activation, repolarizing hPSC-CAR-Ms into tumoricidal macrophages. Additionally, by activating T cells, they enhanced collaborative innate-adaptive responses, amplifying anti-tumor effects (13). Using high-throughput screening, Mukalel et al. (2024) identified oxidized lipid nanoparticles (oLNPs) with innate monocyte tropism and effective mRNA delivery. The optimized C14-O2 oLNP successfully engineered CD19-CAR monocytes *in vivo*, achieving significant B cell depletion, highlighting its therapeutic potential (14). Similarly, numerous novel engineering approaches are being developed, while the efficacy of existing methods continues to undergo constant refinement and enhancement. These ongoing advancements aim to improve the precision, scalability, and effectiveness of current technologies, ensuring they meet the evolving needs of various applications, particularly in the fields of immunotherapy and cellular. Reprogramming of M2 macrophages using a macrophage-directed strategy of delivering CAR and interferon-γ gene (15) is a promising and innovative strategy for improving immune-mediated rejection of cancer (16). In the past, we have amply demonstrated that M1 returned/conditioned macrophages from syngeneic donor mice dictated T cells and promoted immune-mediated rejection of high-grade angiogenic and highly invasive neuroendocrine tumors of the pancreas. Most intriguingly, adoptive transfer of such M1 reprogrammed macrophages (which can be regarded as surrogate CAR-M) potentially normalized tumor vasculature and aided T cell responses against PanNETs (17–19). On the basis of this we believe that HER2/CD47-specific CAR M, while sensing and clearing dead tumor or tumor containing neighboring (immune) cells, also skewed Th1 effector response including priming cytotoxic T lymphocytes (CD8+ T cells), secretion of major Th1 effectors *viz* TNF-α, and IFN-γ & IL-2, and concomitant neutralizing exhaustion markers like PD-1, TIGIT, and LAG-3 on tumor cells (20). This could be explained on the basis of tumor-homing tendencies of macrophages which entitle them as therapeutic cargo to the tumor niche (21). This mechanism might be responsible for the clinical efficacy of HER2-directed CAR-macrophage therapy (CT-0508) under so far known ongoing phase-1 clinical trial (NCT04660929). Most interestingly this trial was safe and tolerable in patients with a variety of solid tumors which indicated efficacy of CAR-M based therapy and raised hope for large variety of tumors in future.

## Challenges and prospective

Although CAR-M-based approaches have several advantages however few bottlenecks are still associated with them. One of the primary issues is that macrophages do not proliferate post-administration, and the amount that patients can tolerate is limited, which can reduce the overall efficacy of interventions. Moreover, exogenous macrophages tend to accumulate in the liver after passing through the lungs, which neutralize their anti-tumor potentials. The high-grade complexity of the human tumor microenvironment over the murine model is another factor that possesses a major challenge for CAR-M therapy to be effective. Such heterogeneous TME restrict the expression of the wide range of tumor antigens, which impact their recognition/binding of TSA/TAA by CAR –M in TME. Thus, whether tumor microenvironment could support or destroy the local CAR-M in the tumor to a tumor-supportive phenotype should be carefully addressed, especially at the clinical research stage. This problem has been observed with CAR-T therapy also and likely to pose a significant obstacle to CAR therapy in general.

Both stability and durability of genetic modifications in macrophages is another hurdle that is associated with off-target effects which can be stabilized by using a CRISPR-based gene editing approach. This can prevent excessive inflammation, which is paramount for maximizing the anti-tumor effects of CAR-M therapies. This can be achieved by floxing iNOS and/or Arginase gene under CD47 promoter to affording palliative potential in iNOs^Floxed^/Arginase^Floxed^CD47+CAR-M to prevent adverse impact. Such strategies would, not only enhance the stability of palliative potential of CAR macrophages but also ensure them to be successful in various clinical trials which would employ these palliative CAR-M. This would enhance the fidelity of the CAR–M program and pave new hope for a wide range of cancer patients.
